# Self-Reported Ability to Walk, Run, and Lift Objects among Older Canadians

**DOI:** 10.1155/2017/1921740

**Published:** 2017-03-06

**Authors:** Jacek A. Kopec, Lara Russell, Eric C. Sayre, M. Mushfiqur Rahman

**Affiliations:** ^1^University of British Columbia, Vancouver, BC, Canada; ^2^Arthritis Research Canada, Richmond, BC, Canada; ^3^Centre for Health Outcomes and Evaluation Sciences, Vancouver, BC, Canada; ^4^East West University, Aftabnagar, Dhaka, Bangladesh

## Abstract

*Aims. *The purpose of the study was to develop new self-report instruments to measure the ability to walk, run, and lift objects and describe the distribution of these abilities among older Canadians.* Methods. *Questions were developed following a focus group. We carried out an online survey among members of the Canadian Association of Retired Persons. The distribution of each ability was described and presented graphically according to age, sex, and number of health conditions. We calculated summary scores for each ability and assessed their reliability and relationships with health status and use of health services.* Results. *22% of the subjects reported difficulty walking 100 m, 15% were unable to run 10 m, and 50% had difficulty lifting 10 kg. Men reported higher abilities than women but differences according to age were small. Test-retest reliability ranged from 0.89 for walking to 0.88 for running and 0.81 for lifting. Scores for the three measures correlated with other measures of health status as expected.* Conclusions*. The study provided new data on self-reported walking, running, and lifting abilities among older Canadians. The new measures are valid, reliable, and easy to interpret. We expect these measures to be useful in clinical and research settings.

## 1. Introduction

Physical activity provides many health benefits, such as lower risk of heart disease and several types of cancer, improved muscle strength and mobility, longer life expectancy, and better self-reported quality of life [1–3]. However, physical activity is often limited among older persons and those with chronic conditions due to physical disabilities. Although there exist a large number of standardized questionnaires to measure physical function [4–7], population data on the distribution of specific abilities, such as walking, running, or lifting, among older adults are somewhat limited. This is partly due to methodological issues in measuring self-reported physical function.

In population surveys, physical function is usually measured using generic or diseases-specific health or quality of life questionnaires. For example, Statistics Canada provides data on the proportion of persons with mobility disability in Canada from the Population and Activity Limitation Survey in which mobility is measured by questions about difficulty in activities of daily living and limitations in the kind or amount of activity a person can do [8]. A 10-item physical function scale is part of the Short Form-36 Health Survey (SF-36), the most commonly used generic health measure [9]. An adaptive, item response theory (IRT) based measure of physical function with a 124-item bank has recently been validated [10]. These multi-item scales combine questions on different aspects or dimensions of physical function, for example, upper and lower body function and activities of daily living, into a single score. Overall scores derived from responses to multiple items covering a wide range of abilities may not be easy to interpret, especially for a clinician unfamiliar with the content of the instrument. In conventional measures, a detailed analysis of responses to each item is possible, but such an analysis is time-consuming and, depending on the items included in the measure, may not provide a comprehensive assessment of, or sufficient detail for, any specific ability. In adaptive testing, the user may not even know which items have been administered.

In this study, we developed self-reported measures of walking, running, and lifting abilities based on the Activity Space Model (ASM) of disability [11]. Ability was conceptualized as a relationship between an objectively defined activity level, expressed in physical units (distance, weight), and subjective perception of difficulty or effort. In this approach, items measuring a given ability are not considered independent. All items pertain to ordered levels of the same activity, defined along a single physical dimension, and ability is represented graphically by a (monotonic) curve. In the ASM, the area under the curve is a reasonable overall measure of a given ability [11]. The main justification for this novel approach is that it may offer some advantages compared with conventional psychometric measures of physical abilities. These advantages include conceptual unidimensionality, improved interpretation, and greater detail and comprehensiveness in the assessment of specific abilities. Our purpose in this article was to demonstrate the usefulness of this approach in measuring physical abilities and describe the ability to walk, run, and lift objects in a large sample of older Canadians.

## 2. Methods 

### 2.1. Instrument Development

Questions pertaining to walking, running, and lifting were developed following a 3-hour focus group with 8 individuals. Participants with arthritis or heart disease residing in Vancouver, Canada, were recruited through newspaper advertisements. In the focus group, the participants completed several previously developed questionnaires, and the pros and cons of different questionnaire layouts were discussed. The final format and wording of the questions are shown in Appendix. We used a matrix format with five response options. For walking, the question stem asked “In your present health, how difficult is it for you to walk the following distances?” Five distances were presented in order from the shortest to the longest, spaced equally on a logarithmic scale (except the last one): 10 m (30 feet), 100 m (1 block), 1 km (10–15 min walk), 10 km (2-hour walk), and 50 km (10–12-hour walk). The response options were “not difficult at all,” “a little difficult,” “somewhat difficult,” “very difficult,” and “unable.” For running, the distances and response options were similar. For lifting, the response options were the same and the questions asked about lifting 6 weights from waist to shoulder level, ranging from 250 g to 100 kg. The weights were also given in pounds and examples of objects were provided for each weight (e.g., a 4-litre milk container or a one-year old child).

### 2.2. Data Collection

We carried out an online survey among members of the Canadian Association of Retired Persons who had previously agreed to receive email requests for participation in research. In addition to the new instruments, the survey questionnaire included an IRT-based computerized adaptive measure of 5 domains of health-related quality of life (CAT-5D-QOL) [12, 13], and questions about overall health, chronic conditions, and use of health services. A randomly chosen subsample of subjects completed the SF-36v2 [14]. The questionnaire was administered on an online survey system developed and hosted at Arthritis Research Canada [12]. All subjects received an introductory email followed by two email reminders. A test-retest was performed on a subsample of the respondents. All subjects consented to participate in the study. The study was approved by the University of British Columbia Behavioral Research Ethics Board.

### 2.3. Scoring and Data Analysis

For each item in the new measures, the difficulty levels were assigned scores from 0 (unable) to 4 (not difficult at all). These scores were converted to percentage scores (0–100%). We plotted individual ability curves, showing the relationship between difficulty and activity levels. Mean ability curves for groups according to age, sex, and number of chronic conditions were derived by calculating the mean difficulty for each level of activity. Summary ability scores for each measure were obtained by summing up the scores for all items, expressed as a percentage of the maximum possible score (0–100%). Higher scores represented higher ability. Scores from the CAT-5D-QOL and SF-36 were norm-based (mean = 50 and SD = 10). They were obtained following established and previously published methods [12, 14] with higher scores denoting better health. Ceiling effect was defined as the proportion of subjects obtaining maximum possible score and floor effect was defined as the proportion with the lowest possible score.

Descriptive data included the frequency of responses to all options on all items. We obtained the distribution plots for the summary scores and calculated means and standard deviations of the scores for each measure. Test-retest reliability of the summary scores was evaluated by calculating the Intraclass Correlation Coefficient (ICC). To determine construct validity, we obtained Pearson's correlations with CAT-5D-QOL and SF-36 domains and assessed the relationships between ability scores and demographic variables, chronic conditions, use of medication, visits to doctors, and hospitalization in the past year in multivariable regression analysis. 

## 3. Results

### 3.1. Characteristics of the Sample

The online questionnaire was completed by 1,089 subjects (response rate 35%). Baseline descriptive data are shown in Table 1. The average age of the respondents was 66.3 years (SD 7.1) and 56% were women. About 54% had college or university education and 29% had high school education or less. 24% reported no health conditions, while 29%, 22%, and 26% reported one, two, and three or more conditions, respectively. About 14% percent did not take any medications in the past 4 weeks while 52% reported taking 3 or more medications. The SF-36 was completed by 549 individuals. Baseline mean physical and mental composite scores (norm-based) in this subsample were 44.3 (range 10.9–65.6) and 53.3 (12.4–71.0), respectively.

### 3.2. Distribution of Abilities in the Study Sample

Frequencies of responses to each question are given in Table 2. Missing data were rare (<5%), except for walking 50 km (9.6%). There was very little ceiling effect for any of the measures. Difficulty in walking 10 m was reported by 14.3%, 100 m by 22.2%, and 1 km by 37.8% of the respondents (excluding missing), while 21.6% had no difficulty walking 10 km. Only 1.3% were unable to walk 100 m. However, 50.0% found it difficult and 15.4% were unable to run 10 m (floor effect). Furthermore, 76.4% had difficulty running 100 m, 24.5% were unable to run this distance, and 50.3% were unable to run 1 km. On the other hand, 24.0% stated they could run 10 km. Very few respondents reported problems lifting weights up to 1 kg, but 24.7% had difficulty lifting 4 kg and 51.1% had difficulty lifting 10 kg (3.1% were unable). On the other hand, 75.0% said they could lift 50 kg (7.3% without difficulty).

**Figure 1:**
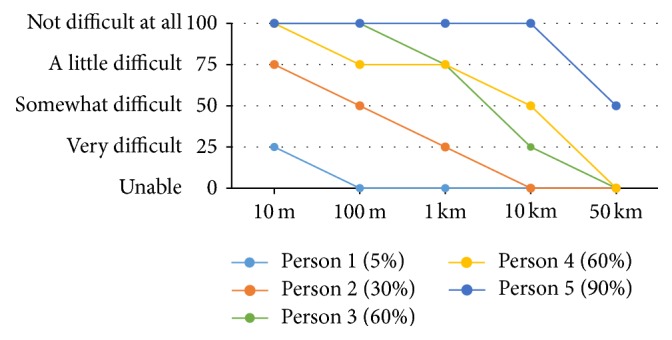
Examples of five individual ability curves: walking ability curves for 5 persons with summary scores ranging from 5% to 90%.

**Figure 2:**
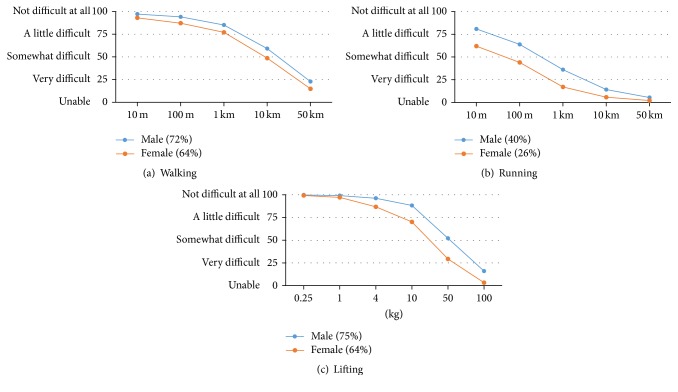
Mean walking, running, and lifting ability curves and summary scores for men and women.

Examples of walking ability curves for persons with varying levels of ability are shown in Figure 1. Mean walking, running, and lifting ability curves for men and women are shown in Figures 2(a)–2(c). The shapes of the curves for walking and lifting are similar, with relatively little difficulty for lower levels of activity, whereas the shape for running is different and characterized by relatively high levels of difficulty. Men reported higher levels of ability than women throughout the full range on all 3 measures, with the largest differences seen for running up to 1 km and lifting 10 kg or more.

**Figure 3:**
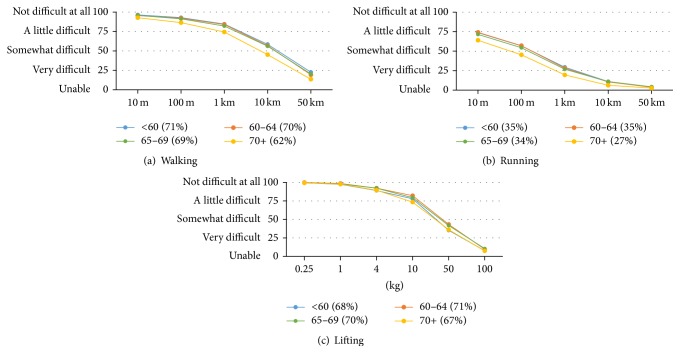
Mean walking, running, and lifting ability curves and summary scores by age group.

Mean ability curves according to age are shown in Figures 3(a)–3(c). The data indicate very little effect of age on walking all distances up to age 70 in this population and somewhat reduced walking ability after age 70. A similar pattern was seen for running, although the ability curves for all age groups were much lower. In terms of lifting, our data showed virtually no difference between the age groups.

**Figure 4:**
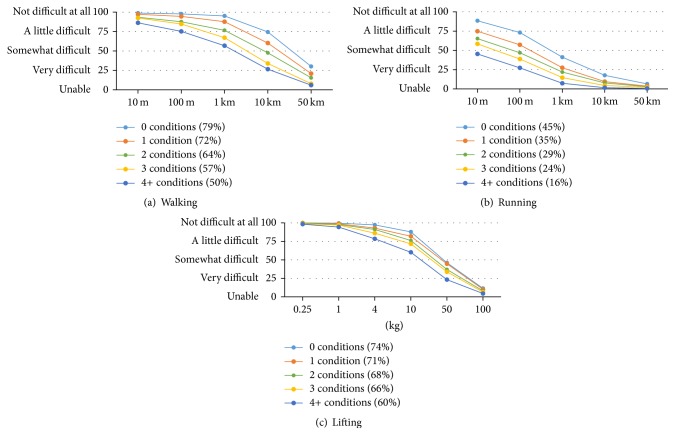
Mean walking, running, and lifting ability curves and summary scores by number of conditions.

The ability to walk, run, and lift objects differed substantially according to the number of chronic conditions reported (0, 1, 2, 3, and 4+), as shown in Figures 4(a)–4(c). The differences were particularly large for walking 1 km and 10 km. For running, the number of chronic conditions was also a strong discriminating factor, mainly for the shorter distances (10 m to 1 km). With respect to lifting, the differences were somewhat smaller but a similar pattern was observed, with greatest differences for lifting 4 kg, 10 kg, and 50 kg.

### 3.3. Measurement Properties of the Summary Scores

Mean summary scores were 67.4% (range 0–100%, SD 21.2) for walking, 32.1% (0–100%, 23.0) for running, and 69.2% (17–100%, 13.6) for lifting. Retests were performed between 1.5 and 4.5 weeks after the baseline (mean 18.3 days, SD 3.4 days). Test-retest reliability (ICC) was high: 0.89 for walking (*n* = 287), 0.88 for running (*n* = 280), and 0.81 for lifting (*n* = 289). Correlations among the new measures were 0.71 (walking and running), 0.49 (walking and lifting), and 0.51 (running and lifting). Summary scores correlated as expected with the CAT-5D-QOL and SF-36 domains (Table 3). The highest correlation was between walking ability and the WALK domain of the CAT-5D-QOL (*r* = 0.87). For lifting ability, the highest correlation with the CAT-5D-QOL domains was with HAND, followed by DAILY, WALK, and PAIN. Among the SF-36 domains (subsample), the strongest correlation for all 3 measures was with physical function and the weakest with mental health (for walking and running abilities) and role emotional (for lifting).

In the regression analysis adjusted for age and sex, lower walking, running, and lifting ability scores were strongly and significantly associated with greater number of conditions and greater use of health services (Table 4). The standardized regression coefficients were highest for walking, followed by running and lifting. For example, the standardized coefficients for walking ranged from −0.45 for the number of chronic conditions to −0.17 for hospitalization.

## 4. Discussion

In this study, we used newly developed and validated questions about physical abilities, based on the Activity Space Model of disability [11], to describe the ability to walk, run, and lift objects in a large community sample of older Canadians. The study showed how the level of difficulty in basic functions, such as walking, running, or lifting, depends on the level of activity (distance or weight). For a broad range of activity levels, the study provides data on the proportion of individuals with various levels of difficulty, including those unable to perform the activity. We also showed how the ability curves can be presented graphically and how they vary according to sex, age, and number of reported chronic conditions. As expected, we found that men reported a higher level of function for all 3 abilities studied, although the differences varied somewhat according to the type and level of activity. However, differences according to age were relatively small, especially for persons <70 years of age. It is the presence and, notably, the number of chronic conditions that showed the strongest discrimination with respect to walking, running, and lifting abilities. We also found a strong relationship between these abilities and the use of health services, such as hospitalization, visits to physicians, and use of medication.

Limitations of the survey include the possibility of coverage error and a relatively low response rate which may lead to selection bias and limit generalizability of the results. However, response rates in the 30–40% range are not uncommon in online surveys of the general population [15]. Our study provided new data that are not easily obtainable or comparable with published data from other population surveys. For example, in the SF-36 questionnaire there are questions about walking 100 yards, several hundred yards, and more than a mile (with 3 levels of limitation each), but they are scored as part of a 10-item physical function scale. Separate questions about running or lifting are not included. The 2006 Participation and Activity Limitation Survey (PALS) in Canada [8] asked about ability to walk (yes/no) and difficulty (some, a lot, and unable) walking 1/2 kilometres, walking up/down a flight of stairs, carrying 5 kg for 10 meters, standing in line for >20 min, standing in one spot for 20 min, and moving from one room to another. While such data may be used to determine the proportion of persons with difficulties performing certain activities at a specified level, they provide a very selective and limited picture of any particular ability.

It should be clear that the proportion reporting a specified level of difficulty depends on the level of activity (e.g., distance, weight). Our definition of ability as a relationships between the level of activity and perceived level of difficulty offers greater clarity with respect to the dimensions being measured. The questions are straightforward to answer while the resultant (monotonic) ability curves should be easy to interpret for a clinician. Compared with a response profile from a typical multi-item instrument, a series of ability curves provides an instant and precise assessment of the key abilities. Another potential advantage of this approach is that translation and cross-cultural equivalence of the instrument may be easier to achieve. For these reasons, we would argue that an ability curve is a more comprehensive, detailed, and easier interpretable measure of ability in a specific functional domain than arbitrarily defined proportion disabled responses to individual questions from a standard health status measure, or even a psychometric physical function score. We expect the approach to measuring physical abilities proposed here to be useful in a clinical setting, where precise, rapid, and highly interpretable assessment of specific abilities is important. Such an assessment may also be important in a research context.

The summary score (interpretable as area under the ability curve) is a convenient overall measure of ability, based on responses to multiple, conceptually unidimensional items. While in our view the summary score should not replace graphical representation of abilities, it is useful for statistical analyses and comparisons. Test-retest reliability of the summary scores was high. The scores correlated as expected with specific domains of established health instruments and discriminated very well according to health services use, which demonstrates their convergent and discriminant validity.

We acknowledge that in some research applications the user may only be interested in an overall physical function score and not need to know which abilities are actually evaluated. Also, some users may prefer to rely on instrument developers to decide which activities to include. For some respondents, answering questions pertaining to objectively defined activity levels may be more difficult than referring to familiar daily activities. Therefore, we included additional clues (e.g., walking time) or examples (e.g., objects of different weight) when describing the levels of activity. Some researchers might perhaps argue that estimating the entire ability curve is inefficient and the amount of detail it provides may not be necessary. However, in the context of measuring specific abilities, it is useful to know the levels of activity (e.g., distance to walk) associated with extreme response options (not difficult at all and unable) because of their impact on participation in more complex activities and social roles, such as work, housework, school, or recreation. The assessment can be made more efficient by applying simple skip logic available in most online survey systems. For example, if the respondent is unable to walk 100 m, there is no need to ask about 1000 m.

The approach to measuring physical abilities presented here could be used to develop a new “gold standard” for measuring physical function. To this end, the ability curve for each ability could be derived by asking participants to perform well-defined activities and measuring the subjective level of difficulty for systematically varied activity levels. Another potential research objective might be a better understanding of the mathematical relationship between activity level and perception of difficulty or effort for various types of activities and various scales of measurement, similar to studies in psychophysics. Finally, self-report measures of other abilities, both elementary (e.g., to climb stairs, stand, bend, and reach) and more complex (e.g., ability to work), could be developed using the methodology presented here and we would encourage this line of research.

## Figures and Tables

**Table 1:** Characteristics of the study population.

Variable	*N*	Mean (range, SD) or %
Age	1072	66.3 (29–87, 7.1)
Sex		
Male	478	44.1
Female	607	55.9
Education		
High school or less	311	28.7
Technical/trade school	189	17.4
College/university	396	36.5
Post graduate	189	17.4
Number of chronic conditions		
None	261	24.0
One	313	28.7
Two	237	21.8
Three or more	278	25.5
Number of medications taken (past 4 weeks)		
None	154	14.2
One	179	16.5
Two	191	17.6
Three or more	561	51.7
Number of doctor visits (past 12 months)		
Never	31	2.9
Once	128	11.8
Twice	205	18.9
3-4 times	379	34.9
5–10 times	252	23.2
More than 10 times	90	8.3
Overnight stay in hospital (past 12 months)	112	10.3
SF-36 Physical Component Score	549^*∗*^	44.3 (10.9–65.6, 10.9)
SF-36 Mental Component Score	549^*∗*^	53.3 (12.4–71.0, 9.5)

^*∗*^Based on a random sample.

**Table 2:** Distribution of responses to walking, running, and lifting ability questions in the study sample (*n* = 1,138).

Level	Not difficult at all	A little difficult	Somewhat difficult	Very difficult	Unable	Missing
Walking						
10 m	973 (85.5%)	107 (9.4%)	39 (3.4%)	13 (1.1%)	4 (0.4%)	2 (0.2%)
100 m	885 (77.8%)	115 (10.1%)	87 (7.6%)	35 (3.1%)	15 (1.3%)	1 (0.1%)
1,000 m	705 (62.0%)	178 (15.6%)	112 (9.8%)	69 (6.1%)	70 (6.2%)	4 (0.4%)
10,000 m	241 (21.2%)	279 (24.5%)	203 (17.8%)	170 (14.9%)	225 (19.8%)	20 (1.8%)
50,000 m	18 (1.6%)	70 (6.2%)	170 (14.9%)	205 (18.0%)	566 (49.7%)	109 (9.6%)
Running						
10 m	561 (49.3%)	188 (16.5%)	116 (10.2%)	85 (7.5%)	173 (15.2%)	15 (1.3%)
100 m	264 (23.2%)	268 (23.6%)	160 (14.1%)	152 (13.4%)	274 (24.1%)	20 (1.8%)
1,000 m	58 (5.1%)	119 (10.5%)	154 (13.5%)	218 (19.2%)	556 (48.9%)	33 (2.9%)
10,000 m	11 (1.0%)	30 (2.6%)	59 (5.2%)	163 (14.3%)	833 (73.2%)	42 (3.7%)
50,000 m	3 (0.3%)	9 (0.8%)	27 (2.4%)	63 (5.5%)	980 (86.1%)	56 (4.9%)
Lifting						
250 g	1113 (97.8%)	17 (1.5%)	1 (0.1%)	0 (0.0%)	1 (0.1%)	6 (0.5%)
1 kg	1059 (93.1%)	58 (5.1%)	13 (1.1%)	2 (0.2%)	0 (0.0%)	6 (0.5%)
4 kg	852 (74.9%)	187 (16.4%)	62 (5.4%)	26 (2.3%)	5 (0.4%)	6 (0.5%)
10 kg	562 (49.4%)	291 (25.6%)	158 (13.9%)	80 (7.0%)	35 (3.1%)	12 (1.1%)
50 kg	82 (7.2%)	206 (18.1%)	275 (24.2%)	285 (25.0%)	282 (24.8%)	8 (0.7%)
100 kg	6 (0.5%)	26 (2.3%)	71 (6.2%)	157 (13.8%)	850 (74.7%)	28 (2.5%)

14 cases with missing data on all ability questions were excluded.

**Table 3:** Correlations of summary scores for walking, running, and lifting abilities with CAT-5D-QOL and SF-36 domain scores.

	Walking	Running	Lifting
Domain of CAT-5D-QOL	*n* = 1082	*n* = 1066	*n* = 1085
WALK	0.87	0.78	0.52
HAND	0.54	0.59	0.68
DAILY	0.74	0.64	0.53
PAIN	0.66	0.57	0.46
FEEL	0.33	0.28	0.29
Domain of SF-36	*n* = 550	*n* = 535	*n* = 552
SF-36 PF	0.81	0.68	0.55
SF-36 RP	0.64	0.50	0.44
SF-36 BP	0.63	0.51	0.44
SF-36 GH	0.53	0.48	0.37
SF-36 VT	0.54	0.48	0.37
SF-36 SF	0.49	0.34	0.37
SF-36 RE	0.31	0.21	0.19
SF-36 MH	0.24	0.20	0.20

Data for SF-36 are based on a random subsample from the study sample.

WALK: walking; HAND: handling objects; DAILY: daily activities; PAIN: pain or discomfort; FEEL: feelings; PF: physical function; RP: role physical; BP: bodily pain; GH: general health; VT: vitality; SF: social function; RE: role emotional; MH: mental health.

**Table 4:** Relationships between summary ability scores and number of conditions, medication, visits to doctors, and hospitalization (standardized regression coefficients).

Variable	Walking	Running	Lifting
	(*n* = 1067)	(*n* = 1051)	(*n* = 1069)
Number of conditions	−0.45	−0.37	−0.30
Frequency of pain medication	−0.44	−0.42	−0.27
Number of medications	−0.41	−0.37	−0.25
Number of visits to doctors	−0.35	−0.30	−0.26
Hospitalization last 12 months	−0.17	−0.13	−0.12

Standardized regression coefficients refer to how many standard deviations a dependent variable will change per standard deviation increase in the predictor variable. Dependent variables are summary ability scores. All variables except hospitalization are treated as continuous. Coefficients are adjusted for age and sex. All *p* values are <0.001.

## Appendix

Item wording for measures of walking, running, and lifting abilities.

### A. Walking

In your present health, how difficult is it for you to walk the following distances:

  10 m (30 feet)
  □ Not difficult at all
  □ A little difficult
  □ Somewhat difficult
  □ Very difficult
  □ Unable
  100 m (1 block)
  □ Not difficult at all
  □ A little difficult
  □ Somewhat difficult
  □ Very difficult
  □ Unable
  1 km (10–15 min walk)
  □ Not difficult at all
  □ A little difficult
  □ Somewhat difficult
  □ Very difficult
  □ Unable
  10 km (2 hour walk)
  □ Not difficult at all
  □ A little difficult
  □ Somewhat difficult
  □ Very difficult
  □ Unable
  50 km (10–12 hour walk)
  □ Not difficult at all
  □ A little difficult
  □ Somewhat difficult
  □ Very difficult
  □ Unable

### B. Running

In your present health, how difficult is it for you to run the following distances:

  10 m (30 feet)
  □ Not difficult at all
  □ A little difficult
  □ Somewhat difficult
  □ Very difficult
  □ Unable
  100 m (1 block)
  □ Not difficult at all
  □ A little difficult
  □ Somewhat difficult
  □ Very difficult
  □ Unable
  1 km (10–15 min walk)
  □ Not difficult at all
  □ A little difficult
  □ Somewhat difficult
  □ Very difficult
  □ Unable
  10 km (2 hour walk)
  □ Not difficult at all
  □ A little difficult
  □ Somewhat difficult
  □ Very difficult
  □ Unable
  50 km (10–12 hour walk)
  □ Not difficult at all
  □ A little difficult
  □ Somewhat difficult
  □ Very difficult
  □ Unable

### C. Lifting

In your present health, how difficult is it for you to lift the following weights from the waist level to the shoulder level:

  250 g or 1/2 lb (glass of water)
  □ Not difficult at all
  □ A little difficult
  □ Somewhat difficult
  □ Very difficult
  □ Unable
  1 kg or 2 lbs (1 litre milk carton)
  □ Not difficult at all
  □ A little difficult
  □ Somewhat difficult
  □ Very difficult
  □ Unable
  4 kg or 8 lbs (4 litre milk container)
  □ Not difficult at all
  □ A little difficult
  □ Somewhat difficult
  □ Very difficult
  □ Unable
  10 kg or 20 lbs (1 year old child)
  □ Not difficult at all
  □ A little difficult
  □ Somewhat difficult
  □ Very difficult
  □ Unable
  50 kg or 100 lbs (large suitcase)
  □ Not difficult at all
  □ A little difficult
  □ Somewhat difficult
  □ Very difficult
  □ Unable
  100 kg or 200 lbs (6 foot person)
  □ Not difficult at all
  □ A little difficult
  □ Somewhat difficult
  □ Very difficult
  □ Unable

## References

[1] Moore S. C., Patel A. V., Matthews C. E. (2012). Leisure time physical activity of moderate to vigorous intensity and mortality: a large pooled cohort analysis. *PLoS Medicine*.

[2] Gillison F. B., Skevington S. M., Sato A., Standage M., Evangelidou S. (2009). The effects of exercise interventions on quality of life in clinical and healthy populations; a meta-analysis. *Social Science and Medicine*.

[3] Bize R., Johnson J. A., Plotnikoff R. C. (2007). Physical activity level and health-related quality of life in the general adult population: a systematic review. *Preventive Medicine*.

[4] Spilker B. (1996). *Quality of Life and Pharmacoeconomics in Clinical Trials*.

[5] MacDowell I., Newell C. (2006). *Measuring Health: A Guide to Rating Scales and Questionnaires*.

[6] Bowling A. (2001). *Measuring Disease: A Review of Disease-Specific Quality of Life Measurement Scales*.

[7] The Patient-Reported Outcomes Measurement Group http://phi.uhce.ox.ac.uk/instruments.php.

[8] Statistics Canada http://www23.statcan.gc.ca/imdb/p2SV.pl?Function=getSurvey&Id=30014.

[9] Ware J. E., Sherbourne C. D. (1992). The MOS 36-item short-form health survey (SF-36). *Medical Care*.

[10] Rose M., Bjorner J. B., Gandek B., Bruce B., Fries J. F., Ware J. E. (2014). The PROMIS Physical Function item bank was calibrated to a standardized metric and shown to improve measurement efficiency. *Journal of Clinical Epidemiology*.

[11] Kopec J. A. (1995). Concepts of disability: the activity space model. *Social Science and Medicine*.

[12] Kopec J. A., Badii M., McKenna M., Lima V. D., Sayre E. C., Dvorak M. (2008). Computerized adaptive testing in back pain: validation of the CAT-5D-QOL. *Spine*.

[13] Kopec J. A., Sayre E. C., Rogers P. (2015). Multiattribute health utility scoring for the computerized adaptive measure CAT-5D-QOL was developed and validated. *Journal of Clinical Epidemiology*.

[14] Ware J., Kosinski M., Bjorner J. B., Turner-Bowker D. M., Gandek B., Maruish M. E. (2007). *User's Manual for the SF-36v2 Health Survey*.

[15] Guo Y., Kopec J. A., Cibere J., Li L. C., Goldsmith C. H. (2016). Population survey features and response rates: a randomized experiment. *American Journal of Public Health*.

